# Microwave-Assisted Preparation of Hierarchical Porous Carbon Aerogels Derived from Food Wastes for Supercapacitors

**DOI:** 10.3390/nano15050387

**Published:** 2025-03-02

**Authors:** Zijun Dong, Tong Li, Xinghe Xu, Yi Chen, Jiemei Fu, Shichang Sun

**Affiliations:** 1 College of Civil and Transportation Engineering, The Underground Polis Academy, Shenzhen University, Shenzhen 518060, China; 2 College of Chemistry and Environmental Engineering, Shenzhen University, Shenzhen 518060, China

**Keywords:** microwave radiation, carbon aerogel, supercapacitor, food waste

## Abstract

Preparing carbon aerogel in an eco-friendly and inexpensive manner remains a significant challenge. The carbon aerogels derived from food waste (FWCAs) with a three-dimensional connected network structure are successfully synthesized using microwave radiation. The as-prepared FWCA-4 (The KOH/C ratio is 4) has a large specific surface area (1470 m^2^/g), pore volume (0.634 m^3^/g), and a high degree of graphitization. Band-like lattice stripes with a spacing of 0.34 nm, corresponding to the graphite plane, are observed. A high specific capacitance of 314 F/g at 1.0 A/g and an excellent capacitance retention (>90% after 10,000 cycles) make the FWCA-4 suitable for high-performance supercapacitor electrode materials. Furthermore, the specific surface area and pore volume of FWCA-4 are larger and the degree of graphitization is higher than in ordinary porous carbon derived from food waste (FWPC). The assembled symmetrical solid capacitor from FWCA-4 exhibits a maximum energy density of approximately 179.9 W/kg in neutral ion electrolytes. Thus, food waste is successfully used to prepare carbon aerogels through a gelation process using microwave radiation. The recycling of waste biomass is achieved, and the results provide insights for the preparation of carbon aerogels using biomass.

## 1. Introduction

Sol-gel materials, as a class of materials with unique structures and a wide range of application prospects [1,2], have attracted much attention due to their excellent porous structure, including a three-dimensional network structure and large specific surface area (SSA). Among them, carbon aerogel (CA) shows great potential for application in several fields due to its lightweight, porous, and highly conductive properties [3]. Compared with ordinary porous carbon materials (PCMs), the porous architecture of CAs is connected and open, which can shorten the transport path for electrolyte ions, ensuring excellent electrical contact. Generally, organic substances such as resorcinol/formaldehyde and cresol/formaldehyde are used as precursors to form CAs through gelation reactions and subsequent high-temperature carbonization [4,5,6]. These processes often involve complex operating steps and toxic organic reagents, which may be expensive. Preparing CAs in an eco-friendly and inexpensive manner remains a significant challenge [7]. Additionally, although CAs have advantages, such as an adjustable pore structure, good mechanical stability, and electrical conductivity, their hydrophobicity and lack of active sites adversely affect their electrochemical performance [5,8]. Heteroatom doping is effective for improving the inherent surface properties of CAs [9]. While biomass materials have the advantages of low-cost, green, and sustainable development, their chemical composition and pore structure have a significant effect on electrochemical performance [3]. Consequently, the preparation of biomass-based CAs using the inherent structure and natural components of biomass has attracted considerable attention in recent years.

CAs can be prepared via direct carbonization at a high temperature using biomass with a natural aerogel structure, omitting the importance of additives or the synthesis route and thus simplifying the preparation steps, where the gelation process is very important [10]. For example, Yuan et al. prepared CAs with a density of 0.048 cm^3^/g through a typical hydrothermal treatment and subsequent activation using winter melon as a raw material [8]. Similar biomasses include succulents, watermelons, and radishes [11,12,13]. Most of these reports are focused on the use of traditional tube furnaces to heat biomass. The heat source of tube furnaces, which convert electrical energy into heat energy, is outside the heated material. Thus, the surface of the heated material is heated first, followed by the internal particles, resulting in uneven heating [14]. Additionally, traditional heat treatment has the disadvantages of a long processing time (1–2.5 h) and high energy consumption. Therefore, as a unique heating method, microwave radiation has received widespread attention. In contrast to traditional heat treatment, in microwave radiation, the electromagnetic fields can rearrange polar molecules. To produce a friction effect, which converts electromagnetic energy into thermal energy, the interference of the thermal motion produced by other interaction force needs to be overcome. The microwave-radiation-induced activation process is effective for producing abundant and uniform micropores, because the microwave radiation realizes the heating and vibration of the heated substance at the molecular level [15,16]. Additionally, under the action of microwave radiation, the dipoles of some O-containing functional groups (O-CFGs) rotate and generate frictional heat. Additionally, the O-CFGs can even absorb microwaves quickly, generating a large amount of heat and significantly increasing the temperature of the heated material. This increases the temperature of localized regions and forms hot spots whose temperatures may be hundreds of times higher than others, which is conducive to the crystal of carbon changing to an orderly structure from a disordered structure, indicating that microwave radiation has the potential to increase the graphitization of heated material. The current microwave heat treatment technology is mainly used to produce biogas and bio-oil via high-temperature pyrolysis. The preparation of PCMs via microwave radiation remains to be explored deeply. Moreover, in most previous studies, ordinary microwave ovens were used as microwave emission sources. These ovens have the following disadvantages: they reduce the thermal energy conversion; have a low final pyrolysis temperature; and make it difficult to control the final temperature, resulting in unsatisfactory structural properties of the obtained carbon materials [15,17]. In comparison, the preparation of a carbon precursor of CAs using biomass via microwave heat treatment has been less frequently reported.

As mentioned previously, using biomass to prepare CAs usually requires a natural gel structure. Because biomass has no natural gel structure, the gelation process is an essential step in the preparation of CAs. For example, biomass rich in cellulose, starch, and other polysaccharides (waste newspaper, starch, and poplar powder) can be used to synthesize CAs through a simple gelation process [13,18,19,20]. Typically, the use of biomass to prepare CAs depends largely on the choice of precursors. In general, it is easier to prepare a CA using biomass with a natural gel structure or simple component because biomass with various functional groups and complex components makes gelation difficult. Moreover, for biomass without a natural gel structure, additional steps are needed to destroy its original structure, and various factors limit the preparation of CAs from biomass. Food waste is generally complex organic waste composed of food residues such as starches, vegetables, and meat fats. The research on the use of food waste to prepare hydrogels attracted our attention [21]. Food waste contains large amounts of starches, cellulose, and lipids. These polysaccharides and lipids are rich in O-CFGs, such as hydroxyl, carboxyl, and amino ether, which can crosslink to form a macromolecular substance with network structures via the graft copolymerization reaction under the appropriate conditions, providing the suitable conditions for the preparation of CAs from food waste. Furthermore, food waste contains abundant N and O; thus, the double doping of N and O is achieved without adding a dopant. Heteroatomic doping can improve the hydrophobicity of the material while increasing the active sites, thereby enhancing its electrochemical performance [22]. The composition of food waste is complex. Using food waste to prepare CAs not only accomplishes resource utilization of waste but is also economically viable.

In this study, a facile and eco-friendly strategy combining the gel process with microwave radiation heat treatment was developed to prepare CAs using food waste. KOH was chosen to be the activating agent. To compare the structural and electrochemical performance of the CAs and ordinary activated carbon, PCMs were also prepared using food waste under the same heat-treatment conditions. The morphology, structural characterization, and chemical composition of the different samples were analyzed. Different samples were used for testing electrochemical performance in a three-electrode system. Finally, the best sample was used to assemble symmetric capacitors, and the electrochemical performance of the capacitors under application conditions was evaluated to verify their practical applicability.

## 2. Materials and Method

### 2.1. Preparation of Carbon Aerogel

The results of a literature survey indicated that there are differences in the yield and composition of food waste generated in different regions. In this work, to ensure the consistency, reproducibility, and representativeness of the results, all of the experimental materials were proportioned according to the typical values for food waste components reported in the literature, and vegetable leaves, white rice, and meat were used to simulate kitchen waste, and all measurements were carried out at least twice to analyze the content of kitchen waste components. An industrial and elemental analysis of food waste is presented in Table 1 [8,23,24].

First, a high-speed blender was used to crush 30 g of food waste into a slurry. The slurry was added to a 250 mL beaker with 120 mL of deionized water. The mixture was heated slowly at 70 °C and stirred for 20 min using magnetic stirring device. Then, potassium persulfate (KPS) (2.0 mmol/L), the acrylic monomer AA (0.005 g), and the crosslinker methylene bisacrylamide (MBA) (0.005 g) were added to the beaker, followed by stirring at 70 °C until a paste formed. Subsequently, the mixture was kept at 70 °C without stirring to complete the polymerization. The obtained gel product was freeze-dried at –45 °C for 48 h. After the drying, the aerogel was obtained.

The magnetic boat with obtained aerogel was heated in a microwave cavity at 700 °C for 5 min at a heating rate of 45 °C/min. The multi-mode microwave heating equipment (CYPY1100C-S, Changyi Microwave Technology, Kaohsiung, China) used in the experiment has a microwave frequency of 2450 ± 20 MHz and an available power of 2 KW. N_2_ was delivered through the whole heating process to ensure an oxygen-free environment. The obtained product was named CA. Then, the KOH and the obtained CA were mixed thoroughly at different mass ratios (KOH/C = 3:1, 4:1, 5:1), followed by drying at 85 °C for 12 h. The dried mixture was then activated in a microwave cavity at 700 °C for 20 min. Finally, 2 M HCl was used to remove the inorganic salts of the collected products. The food-waste-based CA was obtained after being washed with deionized water until neutral. The samples were denoted as FWCA-3, FWCA-4, and FWCA-5, corresponding to the different KOH/C ratios (3, 4, and 5, respectively). For comparison, food-waste-based porous carbon was prepared using the same method that was employed for FWCA-4, omitting the gel process, and was termed FWPC.

### 2.2. Characterization

A field-emission scanning electron microscope (SEM, Osaka, Japan) was used to observe the micromorphology of the samples. The high-resolution image was observed using transmission electron microscopy (TEM, Osaka, Japan). N_2_ absorption–desorption isotherm was analyzed by automatic SSA, pore analysis, and vapor adsorption instrument (BELSORP max, Osaka, Japan), and the specific surface area and pore diameter were determined based on Brunauer Emmett Teller (BET) and density functional theory (DFT) models, respectively. The crystallinity of the carbon materials was analyzed using X-ray diffraction (XRD, Almelo, The Netherlands). The laser source of Raman spectrometer was 514 nm. The details of X-ray photoelectron spectroscopy (XPS, United Kingdom, UK) and of the equipment above were described in our previous study [15].

### 2.3. Electrochemical Measurements

The preparation of working electrode and electrochemical measurements were described in [15].

## 3. Results and Discussion

### 3.1. Morphological and Structural Characterization

**Figure 1:**
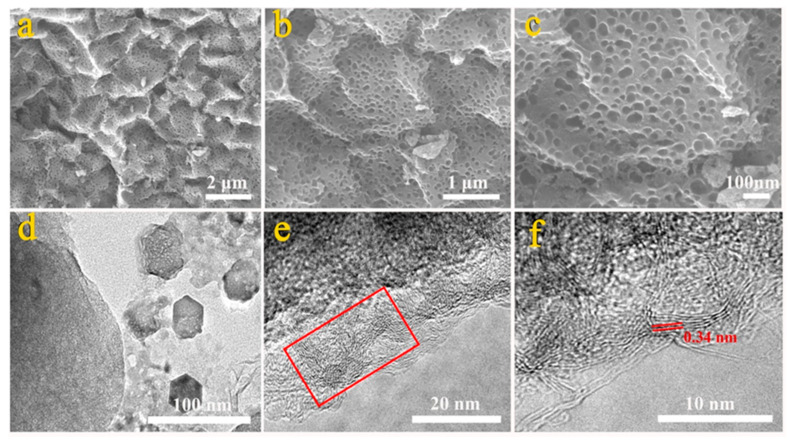
(**a**–**c**) SEM images of different magnifications of FWCA-4. (**d**–**f**) TEM images of different magnification of FWCA-4.

As shown in Figure 1a–c, the FWCA-4 had a uniform and interconnected network pore structure with pore sizes ranging from a few nanometers to tens of nanometers. The macropores functioned as ion buffer pools, and the thin pore walls were conducive to the rapid penetration of ions in the electrochemical behavior [25]. Figure S1 shows an SEM image of the FWPC sample. There were macropores and a few micropores on the FWPC that were not handled by the gelation process. Additionally, the connectivity of the FWPC was poor, the pore-size distribution was not uniform, and the hole wall was thick. Compared with the FWPC, the FWCA-4 sample had a skeletal structure; thus, it had not only a more uniform pore-size distribution but also thinner pore walls. The results indicated that the gel process greatly affected the micromorphology of the carbon products. There were obvious lattice stripes with a lattice spacing of 0.34 nm near the edge of the thin layer of the FWCA-4 sample, indicating the presence of a graphite plane (002) (Figure 1d–f). This suggests that the local regions of FWCA-4 were highly graphitized, which is beneficial to improving the conductivity of electrode materials. The good graphitization performance was attributed to microwave radiation [15,26]. As mentioned previously, microwave radiation heated the material evenly, yielding a uniform micropore structure. This was beneficial to increasing the specific surface area of the material and providing more active sites to participate in electrochemical reactions. Additionally, the dipoles of O-CFGs such as carboxyl and hydroxyl in food waste could rotate under the condition of microwave radiation and then generate heat via the friction effect. The O-CFGs rapidly absorbed the microwaves, resulting in a sharp increase in the local temperature. The temperature of the O-CFGs was generally tens to hundreds of times higher than that of other parts. Localized ultra-high temperatures create hot spots, which enhances the degree of graphitization of the local area of carbon material [27]. Good graphitization significantly increased the electrical conductivity of the material, improving its electrochemical performance in an electrode.

**Figure 2:**
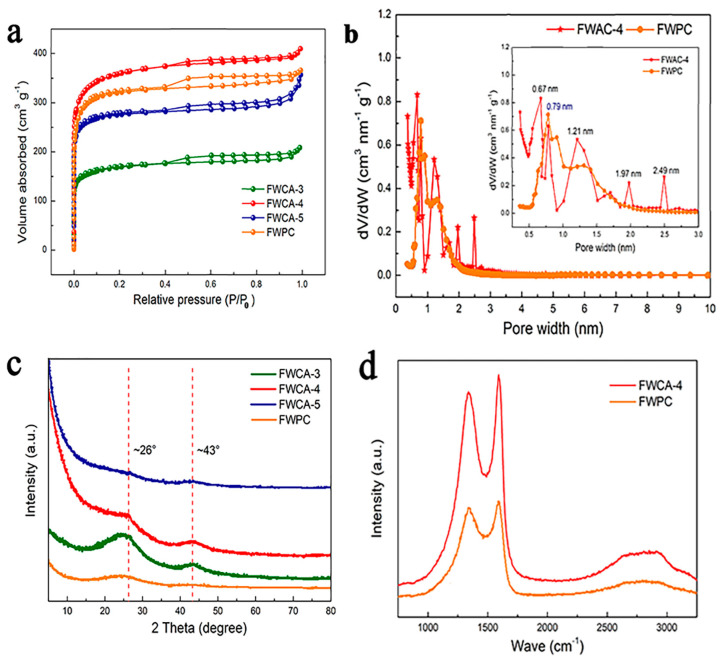
(**a**,**b**) Nitrogen adsorption–desorption isotherms and pore size distributions of FWCAs and FWPC. (**c**,**d**) XRD patterns and Raman spectra of FWCAs and FWPC. N_2_ adsorption–desorption occurred at 77 K.

All of the FWCAs derived from food waste exhibited a typical type I combined with type IV isothermal curve (Figure 2a). The sharp increase in the isotherm at a relatively low pressure (P/P_0_ < 0.1) indicated the presence of micropores. At a relatively moderate pressure, there was a large hysteresis loop (H3), suggesting the presence of mesopores [28,29]. The slight increase at a relatively high pressure suggested that there were macropores in the samples. It was concluded that the FWCAs had a hierarchical porous structure (also referred to as a multimodal porous structure in some studies) comprising micropores, mesopores, and micropore materials. Macropores and mesopores can function as buffer pools for ion transport and channels for rapid ion transfer, respectively. Micropores help to increase the SSA, which facilitates charge accumulation [14,25,30]. The pore–volume distribution and SSA of the samples are shown in Table 2. Among the samples, FWCA-4 had the largest SSA (1470 m^2^/g), which was larger than that of the FWPC (1240 m^2^/g). This high specific surface area could be related to its well-developed microporous structure, which was beneficial to the penetration of electrolytes and the rapid transport of ions. The micropore-to-total-pore volume of the FWPC was 87.5%, which is higher than those of all of the FWCAs. The pore size of the FWPC exhibited obvious polarization: there were micrometer-sized macropores and micropores of <2 nm, indicating that the gel process influenced the morphology of the samples. Combined with Figure 2a, it can be seen that micropores are the main structure of FWCA-4, and a moderate amount of mesopores are also present. The pore size distribution of FWCA-4 indicated the presence of micropores with sizes of 0.67, 1.21, and 1.97 nm, along with mesopores of 2.49 nm (Figure 2b). However, the pore size of the FWPC was mainly concentrated at 0.79 nm. Studies have indicated that a pore diameter (0.36–0.42 nm) that is too small, such as that of micropores (<0.5 nm), might adversely affect the formation of the effective electric double layer [31,32]. Micropores that are too small increase the ineffective SSA without improving the charge storage [31,32]. Appropriate mesopores are conducive to rapid ion transfer at a high current density. However, the FWPC mainly comprised micropores and micron-size macropores. The polarization of the pores was obvious. Although the micropores made a significant contribution to the SSA, which was conducive to the charge accumulation, excessive micropores are not conducive to rapid ion migration. Thus, sample FWCA-4 had a more suitable pore size distribution for the EDLC than the FWPC. The reason for the significant difference between FWCA-4 and FWPC was the addition of the crosslinking agent. The crosslinking reaction connected the disordered molecular chains, forming a macromolecular network structure. The gel process reshaped the internal microstructure of the sample, making the pore structure of FWCA-4 more interconnected and orderly and the pore wall thinner.

The crystallinity of the carbon products significantly affects the electrochemical performance of the electrode material. The FWCAs exhibited high-intensity diffraction peaks at ~26° (Figure 2c), which indicated the presence of graphite carbon [15,26,33]. The low peak at ~43°indicated the presence of graphitic carbon with a disordered or amorphous structure [15,34]. Compared with the FWCAs, the diffraction peak of the FWPC at approximately 26° had a lower intensity and larger width, indicating that the crystal of the FWPC was mainly amorphous. This confirms that the gel process had a favorable effect on the crystallinity of the prepared materials. At the same time, the obtained FWCA had a good degree of graphitization, and high graphitization brought good conductivity and accelerated ion transfer kinetics [35]. A possible reason for this is that the gelation process made the molecules grafted and cross-linked, which increased the degree of order of the molecules while forming new macromolecular substances. The density of the six-membered carbon ring of the molecules increased, increasing the degree of graphitization of the FWCAs. Additionally, as the KOH/C mass ratio increased, the diffraction peaks of the FWCAs became narrower, indicating that the diffraction and crystallinity were enhanced. Research has confirmed that the presence of K^+^ contributes to the transformation of non-graphitizable carbon to graphitized carbon which means that the proportion of sp2 hybrid carbon atoms in carbon materials increases, and the covalent bonds formed by these carbon atoms are beneficial to the conduction of electrons. This is very important for electrochemical energy storage devices. The D band at ~1350 cm^−1^, and G band at ~1580 cm^−1^ indicated the presence of amorphous carbon and graphite interlayer features, respectively (Figure 2d) [36,37]. In particular, the weak peak at ~2700 cm^−1^ might suggest the presence of a typical graphite structure [26]. Furthermore, the *I*_G_/*I*_D_ of FWCA-4 (1.25) was higher than that of the FWPC (1.02), indicating that FWCA-4 had a better degree of graphitization [15].

**Figure 3:**
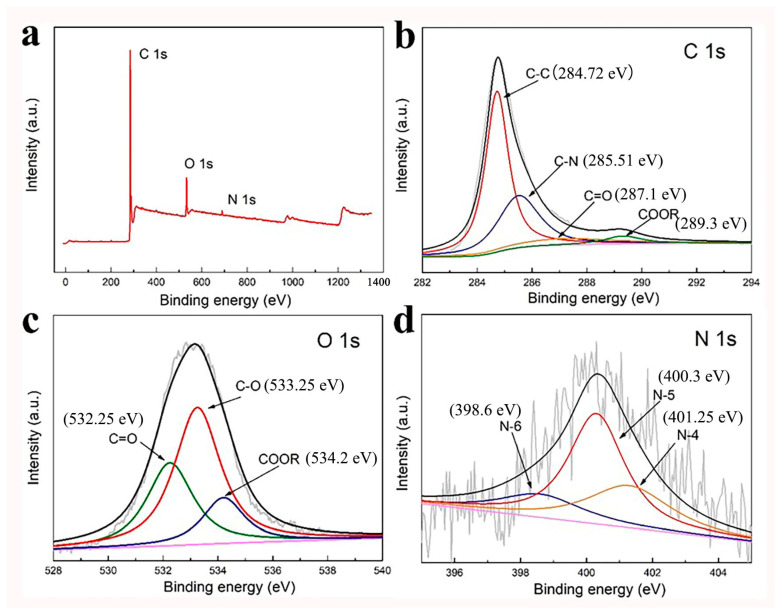
(**a**) XPS survey spectra, (**b**) C 1s, (**c**) O 1s, and (**d**) N 1s of FWCA-4.

The main elements in FWCA-4 were C (89.69%), O (9.31%), and N (0.85%), indicating that the FWCA-4 was successfully doped with O and N. The high-resolution C1s spectrum (Figure 3b) was divided into four peaks, corresponding to C-C (~284.72 eV), C-N (~285.51 eV), C=O (~287.1 eV), and COOR (~289.3 eV) [29]. The O1s spectrum (Figure 3c) exhibited peaks corresponding to three O-CFGs: C-O, COOR, and C=O [15,29]. In the same way, the high-resolution N1s spectrum (Figure 3d) was divided into three peaks: pyridinic (N-6), pyridine (N-5), and quaternary (N-4) [30,38]. Heteroatom doping is conducive to not only improving the hydrophobic properties of the samples but also providing more specific active sites. N atoms are advantageous doping materials because of the unique electronic properties of the lone-pair electrons derived from N and the graphitized conjugated P bonds [39]. N atoms can partially replace C atoms, destroying the carbon skeleton structure and thereby adding more active sites and defects, which enhances the electron density and electron-donating properties [39]. Moreover, the accessible N group can improve the power density of the capacitor, because the N atom in the heterocycle can activate the carbon atom and generate active sites [15,40]. Additionally, N atoms also help to make full use of the exposed surface area of the electrode material to enhance the charge storage [30,41]. Appropriate O-CFGs mainly have two benefits for electrode materials: (1) they can improve the wettability of electrode materials, thereby increasing the accessibility of ions, and (2) the presence of O-CFGs can increase the total specific capacitance of electrode materials by generating pseudo-capacitance [15,29].

**Figure 4:**
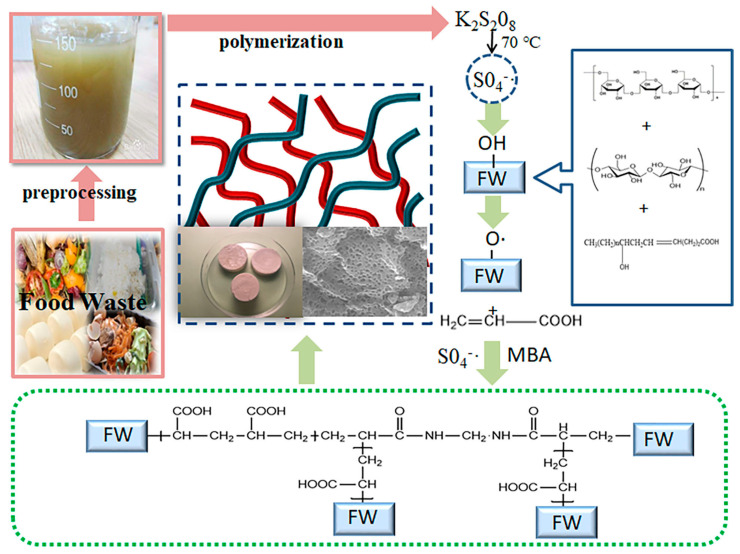
Formation mechanism diagram of FWCAs.

Hydrogel derived from food waste is generated via free-radical graft copolymerization and a crosslinking reaction. The possible formation mechanism is as shown in Figure 4 [21]. The main components of food waste are starch, cellulose, and oil, rich in hydroxyl and carboxyl groups. First, the free radicals produced by KPS extracted H atoms from the hydroxyl groups of the main chains of starch, cellulose, and oil at 70 °C, and active alkoxy groups were formed on the main chain [42]. Subsequently, AA near the active center was initiated by these macromolecular radicals and grafted onto the starch main chain, generating a graft copolymer. Simultaneously, some AA formed a polyacrylic acid (PAA) polymer via polymerization in the presence of MBA and KPS. The presence of MBA promoted the formation of the hydrogel network. Finally, linear acrylic acid (LAA) penetrated the network through H-bonding interactions, forming a semi-interpenetrating network structure.

### 3.2. Electrochemical Performance

#### 3.2.1. Electrochemical Properties in Three-Electrode System

**Figure 5:**
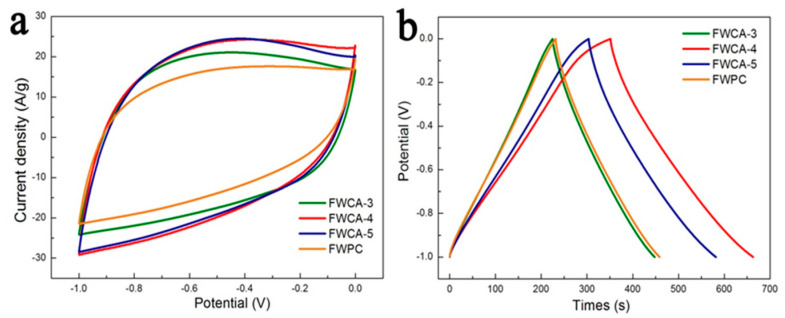
(**a**) The cyclic voltammetry (CV) and (**b**) galvanostatic charge–discharge (GCD) curves of the FWCAs and FWPC. A 6 M KOH electrolyte solution in a three-electrode system was used. The scan rate of the CV curve was 100 mV/s, and the current density measured by the GCD plot was 1 A/g.

As shown in Figure 5a, the four samples all exhibited a good rectangular structure, indicating a stable electrochemical response and ideal electrical double-layer capacitance performance. The CV curves of the FWCAs with the gel reaction were better than that of the FWPC sample without the gel process regarding area and rectangularity, indicating that the gel reaction had a significant positive effect on the electrochemical performance of the carbon materials. This agrees with the aforementioned structure characteristics of the FWCAs. The GCD curves of all of the samples in Figure 5b were triangular, with good symmetry, indicating reversible capacitance and the ideal electrical double-layer behavior [15,17]. Among the samples tested, FWCA-4 had the longest charge–discharge time as well as the largest specific capacitance, indicating excellent electrochemical performance. The specific capacitance decreased in the following order: FWCA-5 > FWCA-3 > FWPC. For the FWCAs, the specific capacitance was proportional to the SSA. Although the SSA of the FWPC was larger than that of FWCA-5, the specific capacitance was smaller, further confirming the positive effect of the gel process on the electrochemical performance of the sample. The electrochemical performance of FWCA-4 and FWPC was analyzed next.

**Figure 6:**
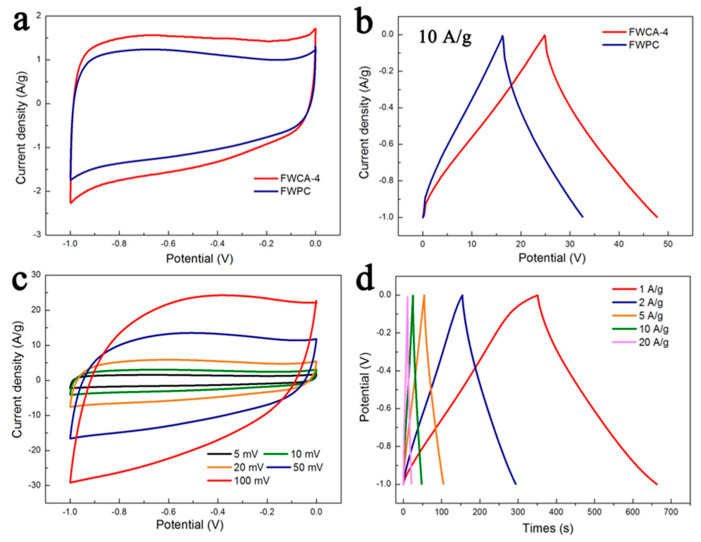
(**a**) The CV of FWCA-4 and the FWPC at a scan rate of 5 mV/s and (**b**) GCD curves at a current density of 10 A/g. (**c**) The CV of FWCA-4 at different scan rates. (**d**) GCD curves of FWCA-4 at different current densities. A 6 M KOH electrolyte solution in a three-electrode system was used.

The CV curves of FWCA-4 obtained at low scan rates exhibited only slight distortion and maintained a relatively good rectangular shape (Figure 6a). Thus, they were superior to those of the FWPC regarding the rectangularity and rectangular area. This indicated that FWCA-4 had the lowest equivalent series resistance, reflecting the good diffusion rate of ions. FWCA-4 had fast ion diffusion and good charge-propagation performance, indicating good electrical double-layer capacitance performance. This may be because its microporous structure is beneficial to the penetration of electrolyte and the rapid transport of ions, which is consistent with the results of BET analysis [7,30]. Although the GCD curves of FWCA-4 and the FWPC maintained a triangular shape at a current density of 10 A/g (Figure 6b), FWCA-4 exhibited a longer charge–discharge time, indicating that FWCA-4 had a higher specific capacitance [17,43]. Additionally, the presence of a small voltage drop (0.05 V) at the beginning of the discharge process suggested that FWCA-4 exhibited a low internal resistance [15]. For FWCA-4, the gel process reshaped the internal microstructure of the carbon materials, making the pore structure of FWCA-4 more orderly and interconnected and making the pore wall thinner. These advantages not only shortened the ion migration path but also made the electrolyte ions more permeable [30]. Additionally, proper mesopore and macropore structures are conducive to ion transport under high-current density conditions. As mentioned previously, owing to the gelation process, FWCA-4 had a better degree of graphitization than FWPC, which is beneficial for improving the conductivity and reducing the internal resistance of the electrode material. In the process of preparing porous carbon from food waste, the gel process played a positive role, significantly affecting the electrochemical performance of the electrode material.

The CV exhibited significant deformation at a scan rate of 100 mV/s (Figure 6c), but maintained good symmetry, indicating that FWCA-4 had fast ion diffusion and good charge-propagation properties [15]. The specific capacitance of FWCA-4 was 314.4 F/g at 1.0 A/g according to the GCD plot shown in Figure 6d. When the current density increased from 1.0 to 20.0 A/g, although the voltage drops at the beginning of the discharging process increased (from 0.008 to 0.15 V), the GCD curve maintained a good triangular structure, indicating that the electrode had good electrical double-layer capacitance behavior and good reversible capacitance.

**Figure 7:**
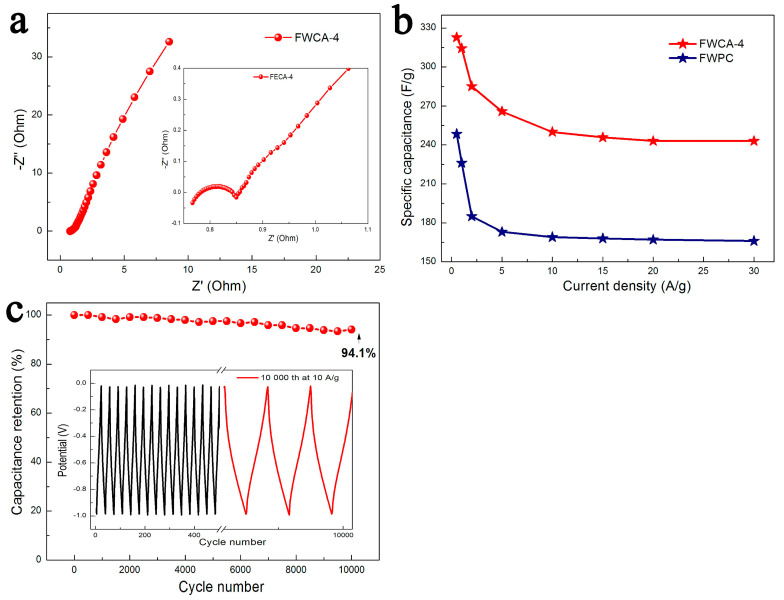
(**a**) EIS plot of FWCA-4 in 6 M KOH solution. (**b**) Specific capacitance of FWCA-4 at different discharge densities. (**c**) Cyclability at current density of 10 A/g over 10,000 cycles. Inset of (**c**) is GCD curve at different cycles. Frequency range was 10 kHz to 10 mHz.

The rate performance of the electrode materials was also discussed in Figure 7b. As the current density increased, the specific capacitance of FWCA-4 decreased, because the diffusion limit of electrolyte ions increased at a high current density. As the current density increased from 0.5 to 20.0 A/g, the specific capacitance of FWCA-4 decreased from 322.8 to 243.0 F/g (a decrease of 24.7%), while the specific capacitance of FWPC decreased by 33.2%, indicating that FWCA-4 had better rate performance. Compared with the FWPC that did not undergo the gel process, the gel process made the pore structure of FWCA-4 orderly and highly connected, yielding better rate performance. Additionally, the pores of the FWPC were unevenly distributed, and the pore walls were thicker. The thinner pore walls of FWCA-4 were conducive to the penetration of electrolyte ions. This might be another reason for its good rate performance.

As the Nyquist plots show in Figure 7a, in the high-frequency range, the X-axis intercept on the real axis (Z’) represents the integrated resistance. The FWCA-4 sample had a low combined resistance of approximately 0.77 Ω. The semicircle in this region reflects the charge-transfer resistance (Rct) [29]. In the mid-frequency, the straight line close to 45° reflects the Warburg diffusion resistance (Rw). A smaller intrinsic resistance indicates higher conductivity, a lower diffusion resistance indicates faster ion transfer, and a lower Rw indicates faster ion diffusion [29]. After 10,000 cycles, FWCA-4 still had a capacitance retention of 94.1% (Figure 7c). Additionally, the GCD curve after 10,000 cycles maintained a good symmetrical triangular structure, and the resistance only increased slightly, indicating good cycle stability.

#### 3.2.2. Electrochemical Properties in Symmetrical System

**Figure 8:**
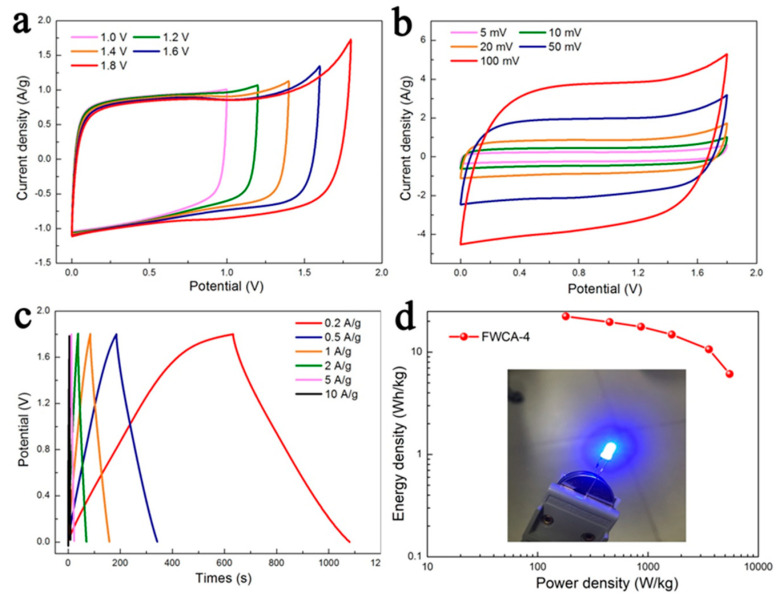
(**a**) CV of FWCA-4 symmetrical supercapacitor at different voltage windows in 1 M Na_2_SO_4_ (aq) electrolyte at scan rate of 5 mV/s. (**b**) CV of FWCA-4 at various scan rates. (**c**) GCD curves of FWCA-4 at different current densities. (**d**) Ragone plot of FWCA-4 related to energy and power densities. Inset of (**d**) is blue LED bulb. Total of 1 M Na2SO4 electrolyte solution was used in symmetrical supercapacitor.

A symmetrical supercapacitor was assembled using FWCA-4 to investigate the electrochemical performance in a 1 M Na_2_SO_4_ electrolyte solution. Neutral electrolytes are able to adapt to a wider voltage window than alkaline and acidic electrolyte solutions [29]. Additionally, the strong solvation of alkali metal cations and sulfate anions in the Na_2_SO_4_ electrolyte is helpful in maintaining the stability of the electrolyte at high voltages [44]. As shown in Figure 8a–d, with an increase in the voltage, the rectangularity of the CV curve of FWCA-4 was deformed only slightly, and good rectangularity was maintained, suggesting that FWCA-4 displayed ideal electrical double-layer capacitance behavior [15,30]. CV curves obtained with different scan rates at a high voltage are shown in Figure 8b. FWCA-4 maintained good rectangularity at high scan rates, indicating that it had good rate performance and ideal electrical double-layer electrochemical behavior. As the current density increased (Figure 8c), the GCD curve of the sample maintained a good symmetrical triangular structure, and the voltage drop was extremely small, confirming that the sample showed a low combined internal resistance, which indicates good electrochemical reversibility as well [29]. The specific capacitance of FWCA-4 was approximately 49.7 F/g at 0.2 A/g. The energy and power density of a symmetric capacitor is shown in Figure 8d. The energy density reached a maximum of 22.35 Wh/kg when the power density was 179.9 W/kg. As the power density increased to 5500.5 W/kg, the energy density remained at 6.1 Wh/kg, which is better than that of ordinary commercial capacitors. Our self-assembled symmetrical capacitors can successfully light blue LED bulbs.

In summary, compared with the FWCA without gel treatment, CAs with the three-dimensional network connection structure (FWCAs) are ideal EDLC electrode materials with excellent electrochemical performance. Firstly, FWCA-4 has a larger SSA and pore volume than the FWPC, which is more helpful for the accumulation of charge and thus contributes to the specific capacitance. Secondly, FWCA-4 has a hierarchical porous structure, which enhances the electrochemical performance of the EDLC at a high current density. However, the FWPC is mainly dominated by micropores and micron-sized macropores, leading to poor rate performance. From the perspective of the crystal structure, FWCA-4 exhibits a higher degree of graphitization than the FWPC, and their *I*_G_/*I*_D_ values are 1.25 and 1.02, respectively. It has been found that gel molecules with a more ordered structure are easier to graphitize [43]. For the foregoing reasons, FWCA-4 displayed superior electrochemical performance to the FWPC, suggesting that the gel process plays a key role in the preparation of CAs as EDLC electrode materials from food waste.

## 4. Conclusions

Food-waste-based CAs (FWCAs) with a three-dimensional network structure were synthesized via a gel process together with a multi-mode microwave pyrolysis furnace and exhibited excellent electrochemical performance as EDLC electrode materials. Compared with the FWPC prepared without the gel process, the as-prepared FWCAs had not only a larger SSA (1470 m^2^/g) and a higher porosity (0.634 m^3^/g) but also a hierarchical three-dimensionally connected porous structure. Furthermore, FWCA-4 had a better degree of graphitization than the FWPC, indicating that the gel process has a positive effect on the degree of graphitization. In terms of electrochemical performance, the specific capacitance of FWCA-4 was 314 F/g at 1.0 A/g. After 10,000 charge–discharge cycles, the capacitance retention rate still remained >90% which showed excellent cycle stability. The maximum energy density reached 179.9 W/kg in an assembled symmetrical solid capacitor in a neutral ionic electrolyte. This forms a solid foundation for its application in practical energy storage devices. Thus, microwave heat treatment was used to prepare CAs from food waste through a gel process. We not only achieved the recycling of waste biomass but also provided new ideas for the preparation of CAs. FWCAs are expected to show a wide range of potential applications in supercapacitors, lithium-ion batteries, and other energy storage applications due to their excellent electrochemical properties and sustainable feedstock sources.

## Figures and Tables

**Table 1:** Properties of food waste.

Proximate Analysis (wt.%)			Elemental Analysis a (wt.%)				
Moisture	Ash ^a^	Volatiles ^a^	C	H	N	S	O ^c^
80.53 ±3.01	15.12 ±0.56	84.88 ±3.24	54.26 ±1.71	8.13 ±0.31	4.65 ±0.13	0.27 ±0.01	32.69 ±2.09

^a^: dry basis ^c^: Subtraction.

**Table 2:** Specific surface area and pore porosity of FWCAs and FWPC.

Sample	S_BET_ (m^2^/g) ^a^	Total Pore Volume (cm^3^/g) ^b^	Micropore Volume (cm^3^/g) ^c^	Mesopore Volume (cm^3^/g)	V_Mi_/V_T_
FWCA-3	771.26	0.512	0.23	0.086	0.449
FWCA-4	1470	0.634	0.40	0.11	0.631
FWCA-5	1070	0.54	0.29	0.14	0.537
FWPC	1240	0.56	0.49	0.07	0.875

^a^: BET surface area; ^b^: total pore volume (P/P_0_ = 0.990); ^c^: volume of micropores, HK method.

## Data Availability

Data are contained within the article and supplementary materials.

## Supplementary Materials

The following supporting information can be downloaded at: https://www.mdpi.com/article/10.3390/nano15050387/s1, Table S1. Comparison of the specific surface area (SSA) and electrochemical performance of FWCA-4 with few previous reported carbon materials-based electrodes. Figure S1. Scanning electron micrograph (SEM) of FWPC. Refs [28,45,46,47,48,49,50,51,52,53] are mentioned in Supplementary Materials.
