# Supplementary material for: Microwave-Assisted Preparation of Hierarchical Porous Carbon Aerogels Derived from Food Wastes for Supercapacitors
Source: Nanomaterials (Basel). 2025 Mar 2;15(5):387. doi: 10.3390/nano15050387 (PMC11901680; doi:10.3390/nano15050387)
Supplement: Supplementary file 1 [file nanomaterials-15-00387-s001.zip › nanomaterials-3424489-supplementary.pdf]

Supporting Information

Tab S1. Comparison of the specific surface area (SSA) and electrochemical performance of FWCA-4 with few previous reported carbon materials-based electrodes.

**Materials**	**Type**	**Form**	**SSA(m^2/g)**	**Electrolyte**	**C_m(F/g)**	**Ref.**
CK11	porous carbon	Flour food wastes	1516	6M KOH	278(0.1 A/g)	1
NHCA-800	Carbon aerogel	cantaloupes	1778	6M KOH	272(1.0 A/g)	2
aCF-6	carbon fiber aerogel	natural cotton	2307	6M KOH	283(1.0 A/g)	3
ACA-SCD	Carbon aerogel	Cellulose microcrystalline	1873	6M KOH	261(1.0 A/g)	4
PGBC	porous graphitic biomass carbon	bamboo	1732	6M KOH	222(0.5 A/g)	5
AHPC	Oxygen-rich porous carbon	Pomelo peel fiber	38.44	6M KOH	222.6(0.5 A/g)	6
LS-800	porous carbon	loofah sponge	1733	6M KOH	304(1.0 A/g)	8
N	CNF/RGO/CNT aerogel	coal	2164	300 μL /cm^2 H2SO4/PVA-gel electrolyte	252(0.5 A/g)	9
N, O-PC-CNTs	N, O co-doped porous carbon nanotubes	coal	2164	6M KOH	287(0.2 A/g)	10
**FWCA-4**	Carbon aerogel	Food waste	**1470**	6M KOH	**314**(1.0 A/g)	**This work.**

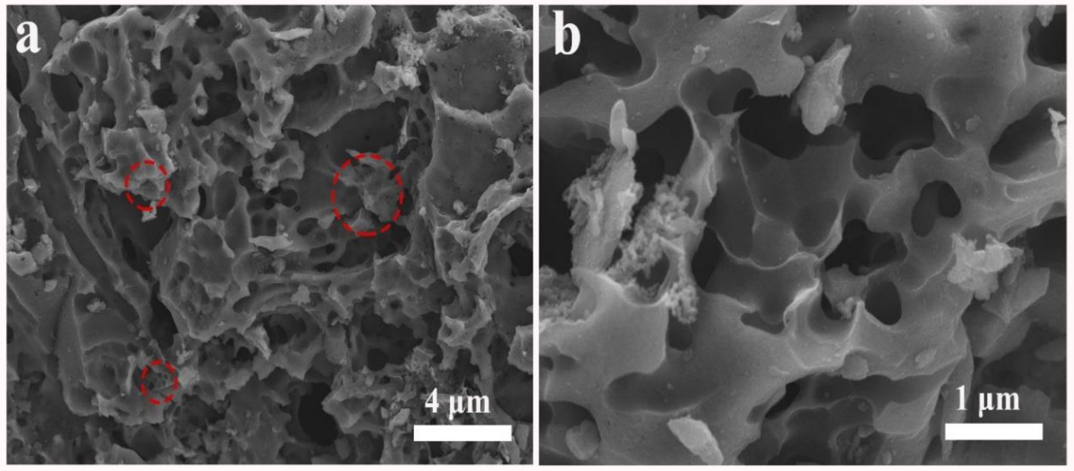

Fig S1. Scanning electron micrograph (SEM) of FWPC.

Fig. S1 shows an SEM image of the FWPC sample. There were ~1-μm macropores and a few micropores on the FWPC that were not handled by the gelation process. Additionally, the connectivity of the FWPC was poor, the pore-size distribution was not uniform, and the hole wall was thick.

References

[1] R. Liu, X. Xi, X. Xing and D. Wu, A facile biomass based approach towards hierarchically porous nitrogen-doped carbon aerogels,Rsc Advances. 6 (2016) 83613-83618. https://doi.org/10.1039/C6RA15185J

[2] Yu. Hang, Liu, Zonghuai, Zhi, Lei, Zhibin, Cheng, Ping and Li,Biomass-Derived Carbon Fiber Aerogel as a Binder-Free Electrode for High-Rate Supercapacitors,Journal of Physical Chemistry C Nanomaterials & Interfaces. 4(2016) 2079-2086. https://doi.org/10.1021/acs.jpcc.5b11280

[3] Wang. Xiaodong, Zu, Guoqing, Shen, Jun, Fang, Yao, Xiandong and Zhang,Nanocellulose-derived highly porous carbon aerogels for supercapacitors, Carbon An International Journal Sponsored by the American Carbon Society. 99(2016) 203-211. https://doi.org/10.1016/j.carbon.2015.11.079

[4] Y. Gong, D. Li, C. Luo, F. Qiang and C. Pan, Highly porous graphitic biomass carbon as advanced electrode materials for supercapacitors,Green Chemistry. 19(2017) 4132-4140.https://doi.org/10.1039/C7GC01681F

[5] J. Li, W. Liu, D. Xiao and X. Wang, Oxygen-rich hierarchical porous carbon made from pomelo peel fiber as electrode material for supercapacitor,Applied Surface Science.416(2017) 918-924.https://doi.org/10.1016/j.apsusc.2017.04.162

[6] S. Gao, X. Li, L. Li and X. Wei, A versatile biomass derived carbon material for oxygen reduction reaction, supercapacitors and oil/water separation,Nano Energy. 33(2017) 334-342. https://doi.org/10.1016/j.nanoen.2017.01.045

[7] Y. Luan, L. Wang, S. Guo, B. Jiang, D. Zhao, H. Yan, C. Tian and H. Fu, A hierarchical porous carbon material from a loofah sponge network for high performance supercapacitors,RSC Advances. 5(2015) 42430-42437. https://doi.org/10.1039/C5RA05688H

[8] Q. Zheng, Z. Cai, Z. Ma and S. Gong, Cellulose Nanofibril/Reduced Graphene Oxide/Carbon Nanotube Hybrid Aerogels for Highly Flexible and All-Solid-State Supercapacitors,Acs Applied Materials & Interfaces. 7(2015) 3263-3271. https://doi.org/10.1021/am507999s

[9] J. Hao, X. Wang, Y. Wang, X. Lai, Q. Guo, J. Zhao, Y. Yang and Y. Li, Hierarchical structure N, O-co-doped porous carbon/carbon nanotube composite derived from coal for supercapacitors and CO2 capture, Nanoscale Advances. 2(2020) 878-887. https://doi.org/10.1039/c9na00761j

[10] C. Zhan, X. Yu, Q. Liang, W. Liu, Y. Wang, R. Lv, Z. Huang and F. Kang,Flour food waste derived activated carbon for high-performance supercapacitors, RSC Advances. 6(2016) 89391-89396.

https://doi.org/10.1039/C6RA18056F
